# Childhood retinol-binding protein 4 (RBP4) levels predicting the 10-year risk of insulin resistance and metabolic syndrome: the BCAMS study

**DOI:** 10.1186/s12933-018-0707-y

**Published:** 2018-05-14

**Authors:** Ge Li, Issy C. Esangbedo, Lu Xu, Junling Fu, Lujiao Li, Dan Feng, Lanwen Han, Xinhua Xiao, Mingyao Li, Jie Mi, Ming Li, Shan Gao, Steven M. Willi

**Affiliations:** 1 Department of Endocrinology, Key Laboratory of Endocrinology, National Health and Family Planning Commission, Peking Union Medical College Hospital, Peking Union Medical College & Chinese Academy of Medical Science, 1 Shuaifuyuan, Wangfujing, Dongcheng District, Beijing, 100730 China; 2 Health Weight Program, The Children’s Hospital of Philadelphia, Perelman School of Medicine at University of Pennsylvania, Philadelphia, PA 19104 USA; 3 Department of Endocrinology, Beijing Chaoyang Hospital, Capital Medical University, Beijing, 100043 China; 4 Department of Biostatistics and Epidemiology, University of Pennsylvania, Philadelphia, PA 19104 USA; 5 Department of Epidemiology, Capital Institute of Paediatrics, Beijing, 100020 China; 6 Department of Endocrinology/Diabetes, The Children’s Hospital of Philadelphia, Perelman School of Medicine at University of Pennsylvania, Philadelphia, PA 19104 USA

**Keywords:** Retinol-binding protein 4, Insulin resistance, Metabolic syndrome, Children, Youth

## Abstract

**Background:**

Elevated retinol-binding protein 4 (RBP4) levels may contribute to the development of metabolic abnormalities, but prospective studies evaluating the association between childhood RBP4 levels and metabolic syndrome (MS) in adulthood are lacking. We investigated whether RBP4 levels during childhood predict cardiometabolic risk at 10-year follow-up.

**Methods:**

The relationships between RBP4 levels, the established adipokines (leptin and adiponectin) and the components of MS were examined in 3445 school-aged children recruited in 2004 for the Beijing Child and Adolescent Metabolic Syndrome study. In 2015, 352 of these individuals completed an in-depth follow-up examination.

**Results:**

Participants with higher childhood RBP4 levels had adverse cardiometabolic profiles at follow-up. Those with incident or persistent MS had higher baseline RBP4 levels than those who never exhibited the elements of MS. Moreover, baseline RBP4 predicted hyperglycemia (OR per SD increase = 1.48, *P* = 0.009), elevated triglyceride (OR = 1.54, *P* < 0.001), elevated blood pressures (OR = 1.46, *P* = 0.015), MS (OR = 1.68, *P* = 0.002) and insulin resistance (OR = 1.44, *P* = 0.015) in the 10-year follow-up phase, independent of baseline BMI. Significant improvements were seen for the net reclassification improvement and integrated discrimination index after adding childhood RBP4 levels into the risk models using conventional cardiometabolic risk factors in predicting MS at follow-up (*P* < 0.05). Leptin and adiponectin demonstrated the expected associations with metabolic disorders.

**Conclusions:**

Childhood RBP4 serves as a risk factor for subsequent development of MS and its components, independent of pediatric obesity. Incorporating childhood RBP4 into conventional cardiometabolic risk assessment models significantly improves the prediction of MS.

**Electronic supplementary material:**

The online version of this article (10.1186/s12933-018-0707-y) contains supplementary material, which is available to authorized users.

## Background

Childhood obesity frequently tracks into adulthood and is a vital contributor to the development of insulin resistance (IR) later in life [1]. Numerous biologically active adipokines, produced by an expanded adipose tissue mass, constitute a pathogenic link between obesity, IR and the metabolic syndrome (MS) [2, 3]. In addition to leptin and adiponectin, which are well-established biomarkers for metabolic dysfunction [2, 4], retinol-binding protein 4 (RBP4), a transport protein for retinols, has been shown to play a role in establishing the link between obesity and IR, and thereby contributes to the pathogenesis of MS and type 2 diabetes (T2D) [5–9].

While liver is the primary source of circulating RBP4, adipocytes become an important secondary source in insulin resistant states [6]. Elevations in RBP4 will induce adipose tissue inflammation and promote systemic IR [10]. Furthermore, RBP4 may play a role in the pathogenesis of T2D by upregulating hepatic expression of the gluconeogenic enzyme phosphoenolpyruvate carboxykinase (PEPCK) and inhibiting insulin signaling in muscle [6]. Conversely, genetic deletion of RBP4 enhances insulin sensitivity [6]. Clinical studies in adults have demonstrated associations between RBP4 levels and obesity, IR, MS and T2D [5, 8, 9, 11], although not all studies agree [12–14]. A limited quantity of cross-sectional pediatric data exhibit an inconsistent relationship between RBP4 levels and childhood metabolic disorders [15–17], while prospective studies in youth remain scarce. In two longitudinal studies of overweight or obese youth, reductions in RBP4 were reported to accompany weight loss, improvement of triglyceride (TG) levels and IR [18, 19], while a small study of Korean boys reported that baseline RBP4 levels did not predict the development of MS over a 3-year follow-up [20]. These existing pediatric studies are significantly limited by their small sample sizes and relatively short follow-up periods.

Leveraging the large cohort within the Beijing Child and Adolescent Metabolic Syndrome (BCAMS) study, we aimed to examine the role of circulating RBP4 in the development of IR, MS and its components from cross-sectional data collected in childhood and longitudinal analysis into young adulthood. In addition, we investigated the incremental value of RBP4 when added to conventional cardiometabolic risk factors in predicting the 10-year risk of cardiometabolic disorders.

## Methods

### Subjects

The BCAMS study is an ongoing cohort study of obesity and related metabolic abnormalities (IR, hypertension, hyperglycemia, and dyslipidemia) as they track from childhood into adulthood in Beijing, China [21]. A representative sample of 19,593 school children, aged 6-18 years, were chosen from four of the eight urban districts and three of seven rural districts in the Beijing area between April and October, 2004. Among these children and adolescents, 4500 were recognized as having risk factors defined by the presence of any one of the following: overweight, total cholesterol (TC) ≥ 5.2 mmol/L, TG ≥ 1.7 mmol/L or fasting glucose (FG) ≥ 5.6 mmol/L based on initial finger capillary blood tests. Moreover, all subjects at increased risk for MS, together with a parallel reference population of 1095 children, were invited to undergo medical examinations for verification based on venipuncture blood samples and clinical examination. Amongst this cohort, 3445 children (50.9% boys, n = 1754) provided blood samples for RBP4 measurement and were thus included in the cross-sectional analysis at baseline. Follow-up studies of this cohort were carried out in 2014. Among the 4500 subjects who were recognized as having risk factors and 1095 normal controls (n = 5595) at baseline, 560 subjects agreed to return to complete the in-depth follow-up examination during the 2 year follow-up period [22]. Among these 560, 352 participants had baseline blood samples available to measure RBP4 levels, and thus were included in the longitudinal analysis. A consort diagram is illustrated in (Additional file 1: Figure S1). The BCAMS study has been registered at http://www.clinicaltrials.gov (NCT03421444).

Normal weight, overweight and obesity were defined by the sex- and age-specific 85th and 95th percentile of BMI, separately, as recommended by the Working Group on Obesity in China Normal weight [23]. The presence of pediatric MS at baseline was defined by the modified criteria of Adult Treatment Panel III (ATP III), which is described in detail elsewhere [24]. MS in adolescents and adults after follow-up was defined by the presence of three or more of the following five components according to the harmonized definition [22, 25]: (1) central obesity: Waist circumference (WC) ≥ 90th percentile for age and gender less than 18 years or WC ≥ 90 cm for adult males and WC ≥ 80 cm for adult females; (2) systolic blood pressure (SBP)/diastolic blood pressure (DBP) ≥ 90th percentile for age and gender for subjects less than 18 years or SBP ≥ 130 mmHg or DBP ≥ 85 mmHg for adults; (3) high-density lipoprotein cholesterol (HDL-C) ≤ 1.03 mmol/L in males, ≤ 1.29 mmol/L in females; (4) TG ≥ 1.70 mmol/L; (5) hyperglycemia: including impaired fasting glucose (IFG), impaired glucose tolerance (IGT), and T2D. The IFG, IGT, and T2D definitions were based on the diagnostic criteria of the American Diabetes Association [26]. Subjects diagnosed with T2D or Type 1 diabetes (T1D) at baseline were excluded from this study. Signed informed consent was obtained from participants and/or parents/guardians. The BCAMS was approved by the Ethics Committee at the Capital Institute of Pediatrics in Beijing. The follow-up study was approved by the Ethics Committee at Beijing Chaoyang Hospital. All the phases of the study complied with the Ethical Principles for Medical Research Involving Human Subjects expressed in the Declaration of Helsinki.

### Clinical and anthropometric measurements

The participants’ height and weight were measured under standardized conditions by trained staff. BMI was calculated as weight (kg) divided by height squared (m^2^). WC was measured midway between the lowest rib and the top of the iliac crest at the end of a normal expiration and the mean value of 3 measurements was recorded. Percent body fat (FAT %) was assessed by bioelectrical impedance analysis (BIA, TANITA TBF-300A). SBP and DBP were measured in the right arm three times, 10 min apart, and the average of the three measurements was used in the analysis. Pubertal development was assessed by Tanner stages (T1–T5) of breast development in girls and testicular volume in boys [27]. This assessment was performed under standardized conditions by two pediatricians match in gender to the child. In addition, questionnaires were used to obtain information on physical activity and dietary intake [28].

### Laboratory measurements

Venous blood samples were drawn after an overnight fast. A 2-h 75 g oral glucose tolerance test (OGTT) was performed in follow-up individuals. The concentrations of plasma glucose (glucose oxidize method), TG, TC, HDL-C and low-density lipoprotein cholesterol (LDL-C) were assayed by the Hitachi 7060 C automatic biochemistry analysis system. HbA1c was assayed using the TOSOH G7 automatic analysis system. Insulin, adiponectin and leptin were measured by monoclonal antibody based enzyme-linked immunosorbent assay (ELISA) [29], which was developed in the Key Laboratory of Endocrinology, Peking Union Medical College Hospital. Insulin assay had an inter-assay coefficient of variation (CV) of < 9.0% and no cross-reactivity to proinsulin (< 0.05%). The intra- and inter-assay CVs were < 5.4 and < 8.5% for adiponectin, and < 7.4 and < 9.3% for leptin, respectively [30]. RBP4 was measured by ELISA kits (Dou set, R&D Systems, Minneapolis, MN, USA) with intra- and inter-assay CVs of 6.2 and 8.5%, respectively. Insulin resistance index was calculated by homeostasis model assessment of insulin resistance (HOMA-IR), HOMA-IR = fasting insulin (mU/L) × FG (mmol/L)/22.5 [31] or insulin sensitive index (Matsuda Index) (ISIM), ISIM = 10,000/(FG × fasting insulin) × (G × I), where G = mean serum glucose, and I = mean serum insulin concentration [32].

### Statistical analysis

All values are expressed as mean ± SD, if not otherwise specified. Variables with skewed distributions, including insulin, HOMA-IR, leptin and adiponectin, were transformed to natural logarithm for analyses. The Chi square test was used for categorical variables. Two-sample *t* test, ANOVA with LSD post hoc comparison test or general linear model with adjustment of confounders were applied to identify differences among continuous variables between two or more groups. Partial correlation analysis, controlling for confounding variables, was used to examine correlations between RBP4 and metabolic parameters. Multivariate logistic regression models were used to estimate ORs for IR, MS and its components. The area under the receiver operating characteristic (ROC) curve (AUC), i.e., *c*-statistic, net reclassification improvement (NRI) and integrated discrimination index (IDI) were used to evaluate the ability and improvement of models to predict MS. The AUCs of various models predicting follow-up MS were compared using Medcalc statistical software version 16.2.0, while NRI and IDI were calculated using R version 3.4.1 (http://cran.r-project.org/). All statistical analyses except NRI, IDI and AUCs comparison were performed using SPSS version 19.0 software for windows (SPSS Inc., Chicago, IL, USA). A *P* value of < 0.05 was considered statistically significant.

## Results

### Findings in the cross-sectional study

A total of 3445 children were included in the cross-sectional analysis at baseline. Among them, 1285 (37.3%) had no MS components, 1737 (50.4%) had 1–2 MS components, and 423 (12.3%) were identified as having MS. Sex- and puberty-related differences of baseline RBP4 levels were evaluated. Subjects were stratified as normal weight (46.4%), overweight (18.6%) or obese (35.0%) based on the age- and gender-specific BMI percentiles. As shown in (Additional file 1: Figure S2), serum RBP4 levels in boys increased steadily with pubertal development in all three weight categories. In normal weight girls, RPB4 increased with pubertal development in parallel with the boys, but exhibited almost no change with pubertal progression in overweight and obese girls. RBP4 levels in boys were higher than in girls at T4 (*P *= 0.045) in the normal weight group, at T4-5 (*P *< 0.001) in the overweight group, and at T3–5 (all *P* <0.01) in the obese group.

The subjects were then divided into non-MS or MS group, and their baseline characteristics were listed in Table 1. As expected, participants with MS showed an adverse cardio-metabolic profile, including higher BMI, WC, FAT%, SBP, DBP, TG, TC, LDL-C, insulin, HOMA-IR and leptin levels, and lower HDL-C and adiponectin levels. RBP4 levels at baseline were significantly higher in subjects who had MS compared with those who did not (RBP4, 38.54 ± 12.43 vs. 33.47 ± 11.19 μg/ml, *P* < 0.001).

**Table 1:** Baseline characteristics of subjects with or without MS at baseline in the cross-sectional study

Variables	Non-MS at baseline	MS at baseline	*P*
N	3022	423	–
Gender (male %)	49.3	61.3	*< 0.001*
Age (years)	11.8 ± 3.1	12.3 ± 2.9	*0.001*
Puberty (stage 1/2/3/4/5%)	32.2/14.4/17.0/ 25.4/11.0	29.5/14.1/18.9/ 24.2/13.4	0.484
Exercise ≥ 3 times/week (%)	56.9	51.4	*0.035*
Dietary score	27.8 ± 4.2	27.1 ± 4.6	*0.001*
Adjusted for baseline age, gender, pubertal stage, physical activity and dietary score			
BMI (kg/m^2^)	21.2 ± 4.5	27.7 ± 4.1	*< 0.001*
WC (cm)	70.5 ± 11.8	87.5 ± 11	*< 0.001*
Body fat percentage (%)	23.5 ± 8.2	31.8 ± 6.9	*< 0.001*
SBP (mmHg)	106 ± 13	121 ± 12	*< 0.001*
DBP (mmHg)	67 ± 10	76 ± 8	*< 0.001*
TG (mmol/L)	0.94 ± 0.46	1.67 ± 0.80	*< 0.001*
TC (mmol/L)	4.08 ± 0.79	4.21 ± 0.84	*< 0.001*
HDL-C (mmol/L)	1.44 ± 0.31	1.11 ± 0.23	*< 0.001*
LDL-C (mmol/L)	2.52 ± 0.72	2.77 ± 0.73	*< 0.001*
Glucose (mmol/L)	5.05 ± 0.47	5.41 ± 0.89	*< 0.001*
Insulin (mU/L)^a^	7.69 (4.81–11.66)	15.09 (10.52–22.43)	*< 0.001*
HOMA-IR^a^	1.74 (1.05–2.67)	3.56 (2.46–5.49)	*< 0.001*
Adiponectin (μg/L)^a^	5.93 (3.98–8.48)	3.88 (2.85–5.47)	*< 0.001*
Leptin (ng/mL)^a^	5.00 (1.72–11.73)	15.2 (8.77–26.84)	*< 0.001*
RBP4 (μg/mL)	33.47 ± 11.19	38.54 ± 12.43	*< 0.001*

All values are reported as mean ± SD, median (interquartile range) or percentage

*BMI* body mass index, *WC* waist circumference, *SBP* systolic blood pressure, *DBP* diastolic blood pressure, *TG* triglycerides, *TC* total cholesterol, *HDL-C* high-density lipoprotein cholesterol, *LDL-C* low-density lipoprotein cholesterol, *HOMA-IR* homeostatic model assessment of insulin resistance, *MS* metabolic syndrome

^a^Variables were ln-transformed before analysis

*P* values were from the Chi square test for categorical variables or the general liner model from continuous variables with adjustment of age, gender, pubertal stage, physical activity and dietary score at baseline

### Relationship of RBP4 with IR, MS and its components in the prospective study

To assess the effect of circulating RBP4 on long-term cardiometabolic risk at follow-up, 352 subjects who underwent an in-depth examination after 10 years were included. Among them, 110 (31.3%) had no MS components, 189 (53.7%) had 1–2 MS components, and 53 (15.1%) were identified as having MS. The characteristics of 352 subjects at baseline and follow-up are listed in (Additional file 1: Table S1). In the partial association analyses (Table 2**)**, baseline RBP4 levels were positively correlated with the follow-up BMI, WC, SBP, DBP, TG, TC, LDL- C, ln-insulin, ln-HOMA-IR and ln-leptin levels, and negatively associated with HDL-C and ln-ISI_M_ (all *P* < 0.05) after adjustment for gender, age, puberty and time of follow-up. Additionally, leptin and adiponectin levels showed similar associations with metabolic profiles after adjusted for the above factors (data not shown).

**Table 2:** Partial correlation coefficients between baseline RBP4 levels and metabolic parameters at the 10-year follow-up

	Coefficients	*P* ^a^
BMI (kg/m)	0.125	*0.027*
WC (cm)	0.132	*0.020*
Fat-mass percentage (%)	0.065	0.248
SBP (mmHg)	0.267	*<* *0.001*
DBP (mmHg)	0.197	*<* *0.001*
TG (mmol/L)	0.231	*<* *0.001*
TC (mmol/L)	0.121	*0.033*
HDL-C (mmol/L)	− 0.114	*0.044*
LDL-C (mmol/L)	0.156	*0.006*
Fasting glucose (mmol/L)	0.082	0.150
OGTT 2 h glucose (mmol/L)	0.085	0.143
HbA1c (%)	0.071	0.200
Insulin (mU/L)^b^	0.119	*0.036*
HOMA-IR^b^	0.124	*0.028*
ISIM^b^	− 0.137	*0.019*
Adiponectin (μg/L)^b^	− 0.060	0.296
Leptin (ng/mL)^b^	0.121	*0.032*
RBP4 (μg/mL)	0.152	*0.007*

*BMI* body mass index, *WC* waist circumference, *SBP* systolic blood pressure, *DBP* diastolic blood pressure, *TG* triglycerides, *TC* total cholesterol, *HDL-C* high-density lipoprotein cholesterol, *LDL-C* low-density lipoprotein cholesterol, *HOMA-IR* homeostatic model assessment of insulin resistance

^a^ *P* values were calculated from partial correlation analysis adjusted for baseline age, gender, pubertal stage and follow-up time

^b^ Variables were ln-transformed before analysis

**Fig. 1:**
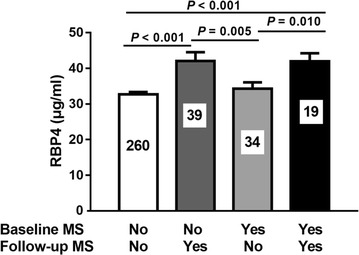
The mean and standard error of serum RBP4 levels at baseline according to metabolic syndrome (MS) status at baseline and follow-up. *P* values were calculated from the general linear model adjusting for age, gender and puberty, physical activity and dietary intake

Among the 299 participants who did not have pediatric MS at baseline, 39 subjects (13.0%) had developed MS at the 10 year follow-up visit, whereas, amongst the 53 subjects with MS at baseline, only 19 subjects (35.8%) were found to still have MS at follow-up. Subjects with incident MS had higher RBP4 levels at baseline than those without MS evident at baseline or follow-up (RBP4, 42.07 ± 14.37 µg/ml vs. 32.73 ± 9.65 µg/ml, *P* < 0.001 after adjustment for gender, age, puberty, physical activity and dietary intake; Fig. 1). Subjects with persistent MS had higher RBP4 levels at baseline than those with baseline MS only (RBP4, 41.94 ± 10.00 µg/ml vs. 34.33 ± 10.15 µg/ml, *P* = 0.010 after adjustment for the same confounding variables).

**Fig. 2:**
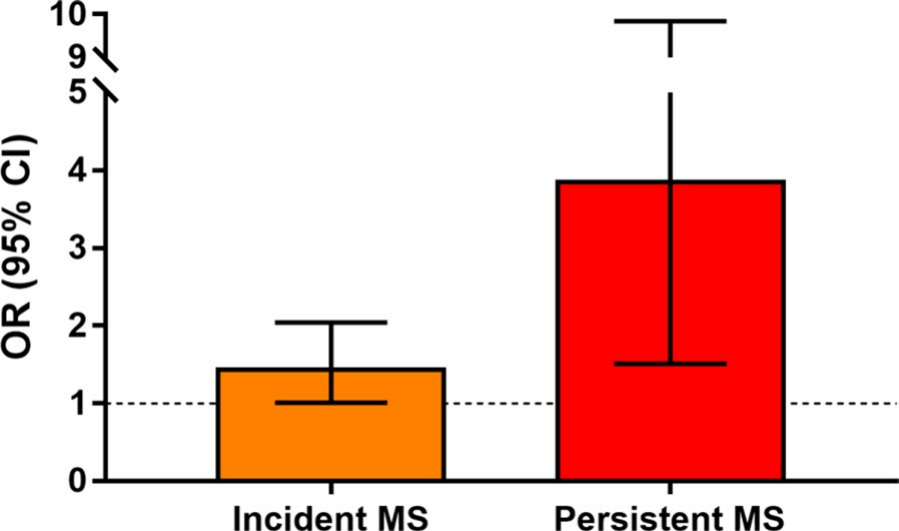
OR (95% CI) for each 10 μg/ml (approximately 1 SD) increase in baseline RBP4 levels for the novel development of metabolic syndrome (MS) in subjects not effected at baseline or the persistence of MS among subjects with MS at baseline and at 10-year follow-up. ORs were adjusted for age, gender, pubertal stage, waist circumference, systolic blood pressure, HDL-C, fasting blood glucose, and TG levels at baseline

We further used multivariable logistic regression to relate baseline RBP4 levels to the risk of having MS at follow-up (Table 3; Fig. 2). To separately explore the potential roles of RBP4 in the development of MS or the tracking of MS from childhood to adulthood, subjects were divided into two groups: non-MS and MS at baseline. In the subjects without MS at baseline, we found that an elevated RBP4 level at baseline was an independent predictor for incident MS upon follow-up (OR per 10 μg/ml (approximately 1 SD at baseline) increase 1.68, [95% CI 1.22–2.34], *P* = 0.002 after adjustment for age, gender, puberty, BMI, physical activity and dietary intake at baseline; Model 2; Table 3; Fig. 2). In addition, adjustment for MS component levels at baseline didn’t change the association between childhood RBP4 levels and incident MS. Moreover, although increased leptin levels and reduced adiponectin levels also predicted follow-up MS (all *P* < 0.05), further adjustment for baseline leptin and adiponectin didn’t modify the associations between baseline RBP4 and follow-up MS (*P* = 0.003; Model 4).

**Table 3:** Baseline RBP4 levels predicts the incidence and persistence of MS in the 10-year prospective study

	The incidence of MS^a^		The persistence of MS^b^	
	OR/unit (95% CI)	*P*	OR/unit (95% CI)	*P*
Model 1	1.77 (1.29–2.43)	*< 0.001*	2.19 (1.14–4.20)	*0.018*
Model 2	1.68 (1.22–2.34)	*0.002*	2.32 (1.18–4.45)	*0.015*
Model 3	1.43 (1.01–2.04)	*0.049*	3.85 (1.51–9.84)	*0.005*
Model 4	1.66 (1.19–2.30)	*0.003*	2.28 (1.06–4.93)	*0.036*

OR indicates odds ratio for per 10 μg/ml (approximately 1 SD) increase in baseline RBP4 levels

*BMI* body mass index, *TG* triglycerides, *HDL-C* high-density lipoprotein cholesterol, *MS* metabolic syndrome

^a^ Excludes subjects with MS at baseline

^b^ Excludes subjects without MS at baseline

Model 1: adjusted for age, gender, and pubertal stage at baseline

Model 2: Model 1 + additionally adjusted with physical activity, dietary score and BMI at baseline

Model 3: Model 1 + additionally adjusted with waist circumference, systolic blood pressure, HDL-C, fasting blood glucose and TG levels at baseline

Model 4: Model 1 + additionally adjusted with adiponectin, leptin levels and BMI at baseline

In the group having MS at baseline, we found that an elevated RBP4 level at baseline was also associated with an increased risk of presistent MS at follow-up (OR 3.85, [95% CI 1.51–9.84], *P* = 0.005; Model 3; Table 3) independent of conventional metabolic risk factors at baseline. Additionally, adjustment for baseline leptin and adiponectin didn’t modify the associations between baseline RBP4 and persistent MS (P = 0.036; Model 4).

With respect to the key elements of MS, after adjusting for age, gender and puberty (Model 1, Table 4), RBP4 levels at baseline predicted IR (OR per 10 μg/ml (approximately 1 SD) increase 1.42, [95% CI = 1.08–1.86], *P* = 0.011), hyperglycemia (OR 1.49, [95% CI 1.11–1.99], *P* = 0.008), elevated blood pressure (OR 1.38, [95% CI = 1.07–1.76], *P* = 0.012) and elevated TG (OR 1.83, [95% CI = 1.40–2.41], *P* = 0.004) at follow-up. Further adjustment for life-style factors, BMI and MS component levels at baseline did not significantly attenuate the associations of RBP4 levels with follow-up IR, hyperglycemia, elevated TG or elevated blood pressure (Model 3) (*P* < 0.05).

**Table 4:** Baseline RBP4 levels predicts IR and MS components after 10-year follow-up

	Number of cases/total number of subjects	Model 1		Model 2		Model 3		Model 4	
		OR/unit (95% CI)	*P*	OR/unit (95% CI)	*P*	OR/unit (95% CI)	*P*	OR/unit (95% CI)	*P*
IR (HOMA-IR ≥ 3.0)	73/352	1.42 (1.08–1.86)	*0.011*	1.44 (1.07-1.92)	*0.015*	1.36 (1.02–1.81)	*0.039*	1.35 (1.01–1.81)	*0.049*
Central obesity	152/352	1.20 (0.95–1.50)	0.125	1.25 (0.99-1.57)	0.065	1.00 (0.74–1.35)	0.979	1.00 (0.74–1.34)	0.974
Elevated blood pressure	75/352	1.38 (1.07–1.76)	*0.012*	1.46 (1.08-1.99)	*0.015*	1.38 (1.01–1.91)	*0.049*	1.33 (1.01–1.76)	*0.046*
Reduced HDL-C	58/352	1.08 (0.80–1.47)	0.612	1.05 (0.77-1.45)	0.756	1.09 (0.78–1.52)	0.609	1.04 (0.75–1.43)	0.830
Elevated TG	61/352	1.83 (1.40–2.41)	*< 0.001*	1.76 (1.33-2.33)	*< 0.001*	1.54 (1.14–2.07)	*0.004*	1.70 (1.28–2.26)	*<* *0.001*
Hyperglycemia	44/352	1.49 (1.11–1.99)	*0.008*	1.48 (1.10-1.98)	*0.009*	1.55 (1.14–2.09)	*0.005*	1.51 (1.12–2.03)	*0.007*

OR indicates odds ratio for per 10 μg/ml (approximately 1 SD) increase in baseline RBP4 levels

*BMI* body mass index, *HOMA-IR* homeostatic model assessment of insulin resistance, *IR* insulin resistance, *TG* triglycerides, *HDL-C* high-density lipoprotein cholesterol, *MS* metabolic syndrome

Model 1: adjusted for age, gender and pubertal stage at baseline

Model 2: Model 1 + additionally adjusted with physical activity, dietary score, and baseline BMI (except central obesity model)

Model 3: Model 2 + additionally adjusted with levels of the individual components at baseline

Model 4: Model 1 + additionally adjusted with adiponectin, leptin levels and BMI at baseline

### Improvement of risk model combining RBP4 levels and MS components in predicting follow-up MS

We next evaluated the value of RBP4 in predicting follow-up MS beyond the information provided by baseline levels of MS components. We used three statistical measures (AUC, NRI, and IDI) to assess the performance of the present risk prediction models. The AUC for the model including baseline age, gender, puberty, WC, SBP, HDL-C, TG, glucose levels and follow-time was 0.785 (95% CI 0.716–0.853). Adding RBP4 levels to the models including MS components increased the AUC (0.813, 95% CI 0.747–0.880) with marginal significance (*P* = 0.062). However, using the more sensitive statistical measures of NRI and IDI, significant improvement was seen between models with and without RBP4 levels. The continuous NRI for adding RBP4 levels was 0.376 (95% CI 0.083–0.669, *P* = 0.012), suggesting that the percentage of individuals with MS correctly classified upward and those without MS correctly classified downward was 37.6% using a model adding RBP4 compared with the initial model including only MS components. The IDI was 0.046 (95% CI 0.011–0.082, *P* = 0.011), indicating that the difference in average predicted risks between individuals with and without the outcome increased significantly when RBP4 was included in the prediction model.

## Discussion

In this cohort study of Chinese youth, high levels of childhood RBP4 at baseline were associated with an adverse cardiovascular risk profile at baseline and upon 10-year follow-up. The most striking, novel finding of the present study is that RBP4 levels measured in childhood were strong predictors of the subsequent development of MS and each of its components (including IR, hyperglycemia, hypertension and hyperlipidemia) 10 years later, independent of obesity. In addition, our data are among the first to confirm the tracking of MS in Chinese children, as has been demonstrated in a number of other cohort studies [33]. Finally, the prognostic value of baseline RBP4 levels led us to uncover the first epidemiological evidence that incorporating childhood RBP4 levels into a risk assessment model using MS components significantly enhances the prediction of subsequent development of MS. Our study also replicated well-known associations between leptin, adiponectin and metabolic disorders.

### Sex-, puberty- and weight status-related differences in RBP4 levels at baseline

As a result of the wide range of ages found in our relatively large sample of Chinese children, we were able to assess pubertal influences on RBP4 and found that this effect was modified by adiposity and differed by gender. We noted that RBP4 levels in boys increased consistently across puberty regardless of their weight status; whereas in girls, this increase in RBP4 across puberty was only evident in the absence of obesity, suggesting a sexual dimorphism in RBP4 levels with regard to obesity. Although the effect of puberty on RBP4 has previously been examined in three small, cross-sectional studies of European and Korean children, those findings were inconsistent; likely due to the small sample size (n < 130) in each [34–36]. Our findings contribute significantly to the limited data available on RBP4 levels in puberty, and thereby allow us to identify which age stage and degree of adiposity might determine RBP4-associated pathologic events and expand our understanding of the pubertal influences on metabolic disorders. Although the precise mechanism is unclear, this finding suggests that any evaluation of the importance of RBP4 in metabolic disorders of children should take gender and puberty into account.

### Relationship of RBP4 with IR and hyperglycemia at follow-up

Our data identify a longitudinal association between circulating RBP4 levels and IR, as well as hyperglycemia. Animal studies have demonstrated RBP4’s pivotal role in IR [10]. Consistent with these experimental observations, childhood RBP4 levels exhibited a reverse association with insulin sensitivity at baseline and were predictive of IR at 10-year follow-up in our study. As IR predisposes one to develop T2D, we found that subjects with the highest levels of RBP4 in childhood had the greatest risks of developing hyperglycemia during the 10-years follow-up phase, although there was a very low incidence of T2D after just 10 years of follow-up (at a mean age of 20.2). Further adjustment for baseline BMI did not change the longitudinal association between RBP4 and dysglycemia, suggesting that this association is not merely a reflection of childhood obesity. Consistent with our results, several adult studies have found strong associations between IR, T2D and elevated RBP4 levels [5, 8, 11], while others fail to endorse these relationships [12–14]. However, the current study presents some of the only prospective pediatric data on RBP4 levels and their association with IR aside from a few cross-section studies with relatively small sample size [15, 16, 34, 37]. The other existing longitudinal data are from rather small studies, but were nonetheless, consistent with our findings [18, 20]. Therefore, our long-term prospective study provides substantial evidence supporting the vital role of RBP4 during childhood in the determination of dysglycemia early in life.

### Relationship of RBP4 with MS at follow-up

The most significant finding of this study is the identification of RBP4’s potential role in the tracking of childhood MS. Nearly 36% of children with MS showed persistence of this condition at 10 year follow-up. Although this is lower than what is found in previous studies [34, 38, 39], it is difficult to compare results across studies due to the inconsistent definition of MS applied. Nonetheless, we believe this is the first study to examine the tracking of MS from childhood to adulthood in a Chinese population [40]. The significance of RBP4 is highlighted by the observations that RBP4 elevations in childhood were present in both those with incident MS at follow-up, as well as those with MS which persisted over 10 years. Of the many possible pathophysiological mechanisms involved in the development and maintenance of MS, we believe that RBP4 is a reliable indicator of innate IR [10], and plays an important role, given its longitudinal association with IR and its ability to predict the onset and persistence of MS after 10-year follow-up. In contrast to a study in seventy overweight/obese adolescent girls from Eastern Europe [41], it is worthy to note that even after adjusting for obesity and the components of MS at baseline, childhood RBP4 levels still predicted MS and its components in our study, suggesting an independent role for RBP4 in the development of MS. Moreover, although the value of other adipokines (e.g., leptin and adiponectin) has been previously established [2], after adjusting for the contribution of adiponectin and leptin, childhood RBP4 levels still predicted MS and its components. Taken together, these are the first prospective data providing evidence that increased RBP4 levels in early childhood confers a risk for not only the development but also the persistence of MS into adulthood.

### Improvement of risk model combining RBP4 levels and metabolic risk factors in predicting MS

Despite strong evidence of the association between RBP4 and metabolic abnormalities, there are limited data on the predictive value of circulating RBP4 for the development of MS. Moreover, previous studies did not evaluate the additional predictive ability of RBP4 beyond the information based on MS components at baseline. In our study, we performed three measures (AUC, NRI, and IDI) to estimate the practical value of RBP4 in addition to components of the MS. While the traditional MS components at baseline strongly informed the prediction of MS at 10 years follow-up in our study, adding childhood RBP4 levels to these risk factors still significantly improves the ability to predict MS at follow-up, as indicated by the continuous NRI and the IDI. It should be noted, that NRI and IDI appear to be more sensitive than the AUC, particularly when risk factors with strong associations to the outcome have already been included in the initial model [42]. As a result, the improvement of AUCs showed a marginal significance in this study. However, there are still limitations and controversies surrounding these two measures, including the possibility of miscalibration of prediction models [43]. Nonetheless, to our knowledge this is the first prospective cohort study to indicate that circulating RBP4 may have intrinsic value in screening for cardiometabolic risk during childhood. Future studies are needed to further delineate specific risk scoring tools and clinical thresholds for predicting MS using serum RBP4.

### Strength and limitation

The major strength of our study is the assembly of a large, well-characterized cohort of subjects with a range of metabolic traits and covariates. The wide array of phenotyping information allows us to adjust for many potential confounders known to correlate with both cardiometabolic risk and RBP4 levels, ensuring a robust analysis. Nevertheless, our study has several potential limitations. Firstly, we could not avoid the possibility of introducing selection bias, as the number of subjects returning for follow-up evaluation after 10 years was relatively small compared to the original cohort, mainly due to enhanced migration patterns among school-aged children in Beijing, and the inherent difficulties of tracking subjects from childhood into adulthood. Nonetheless, the salient characteristics of subjects available for follow-up was similar to the overall cohort at baseline, although BMI, WC, TG, HOMA-IR and leptin were slightly higher than in those lost to follow-up (Additional file 1: Table S2). As these are among the only longitudinal data on RBP4 across childhood, further prospective studies with larger sample size from diverse pediatric populations are warranted to validate our findings. Secondly, although we have taken into account numerous confounding factors in a variety of statistical models compared to previous studies, we could not adjust for all potential confounders, such as kidney function. An adult study (n = 230) and a small pediatric study (n = 85) was reported that kidney function was associated with circulating RBP4 levels [44, 45], but we didn’t measure kidney function of children at baseline. This may be a possible limitation, although no significant association between serum creatinine and RBP4 levels was observed at follow-up after adjustment for age and gender (*P *> 0.05, data not shown). Thirdly, due to the relatively small number of subjects with MS in the follow-up phase, we chose to adjust our logistic regression models for a limited number of important covariates in a single model, in order to avoid introducing artifact by “over adjusting” the model.

In conclusion, in this young Chinese population, circulating RBP4 levels predict the development of MS and its components, independent of pediatric obesity. Adding RBP4 levels to MS components improves the ability to assess the risk of incident and persistent MS over a 10-year period. Thus, our prospective cohort study provides new insight into the potential role of RBP4 as an early biomarker of MS, and may constitute a future target for interventions.

## Additional file

**Additional file 1: Figure S1.** Flow chart. **Figure S2.** Gender- and puberty- differences in serum RBP4 levels. **Table S1.** Baseline and follow-up characteristics of subjects in the prospective study. **Table S2.** Baseline characteristics of subjects with or without loss to follow-up.

## Abbreviations

BMI: body mass index
DBP: diastolic blood pressure
FBG: fasting blood glucose
HDL‑C: high‑density lipoprotein cholesterol
HOMA-IR: the index of homeostasis model assessment of insulin resistance
IDI: integrated discrimination index
IFG: impaired fasting glucose
IGT: impaired glucose tolerance
ISIM: insulin sensitive index (Matsuda Index)
LDL-C: low-density lipoprotein cholesterol
MS: metabolic syndrome
NRI: net reclassification improvement
OGTT: oral glucose tolerance test
ROC: receiver operating characteristic
SBP: systolic blood pressure
T2D: type 2 diabetes
TC: total cholesterol
TG: triglycerides
WC: waist circumference
