# Supplementary material for: Childhood retinol-binding protein 4 (RBP4) levels predicting the 10-year risk of insulin resistance and metabolic syndrome: the BCAMS study
Source: Cardiovasc Diabetol. 2018 May 14;17:69. doi: 10.1186/s12933-018-0707-y (PMC5950249; doi:10.1186/s12933-018-0707-y)
Supplement: Supplementary file 1 — Additional file 1: Figure S1. Flow chart. Figure S2. Gender- and puberty- differences in serum RBP4 levels. Table S1. Baseline and follow-up characteristics of subjects in the prospective study. Table S2. Baseline characteristics of subjects with or without loss to follow-up. [file 12933_2018_707_MOESM1_ESM.docx]

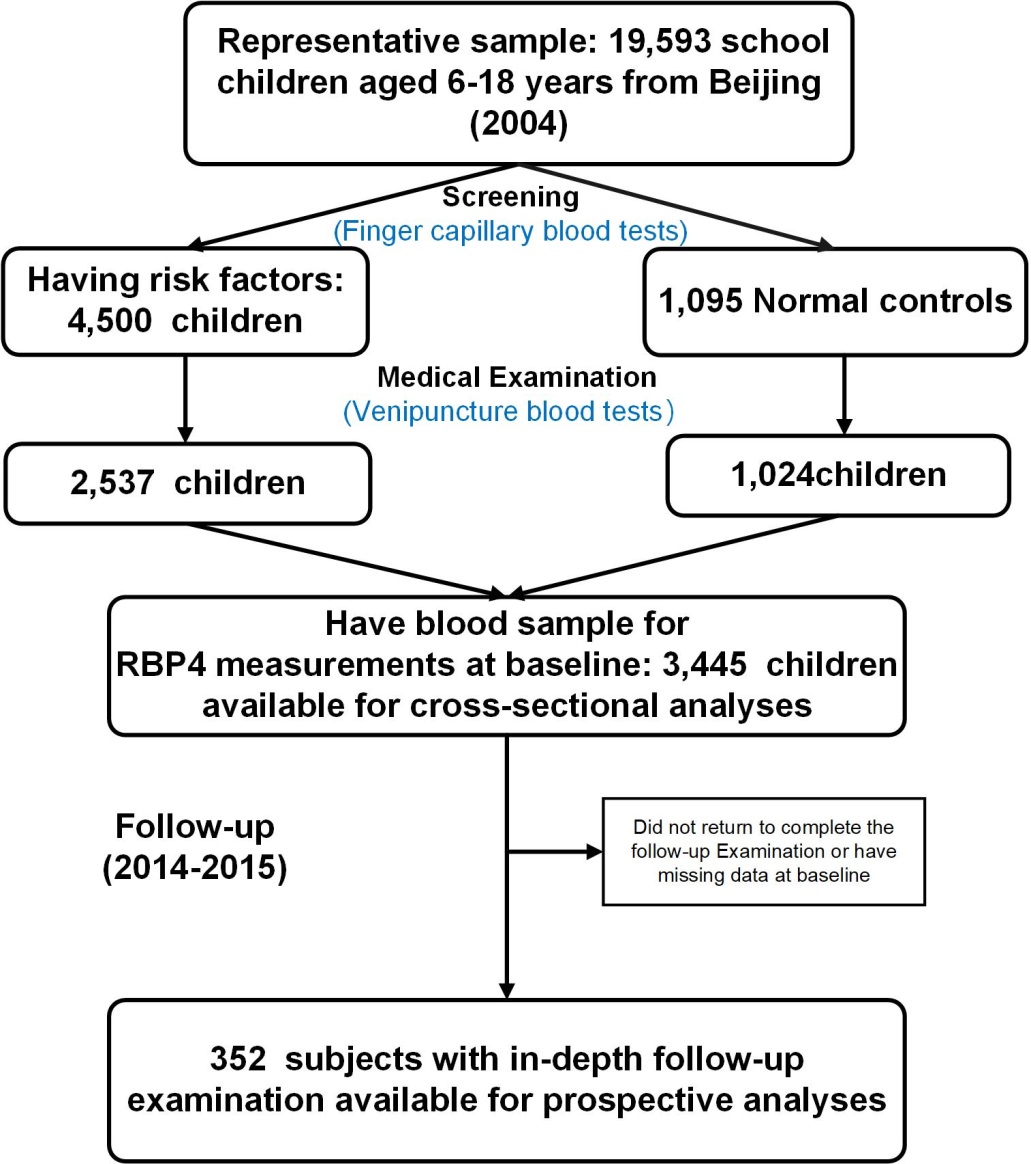


**Fig. S1 Flow chart**

**
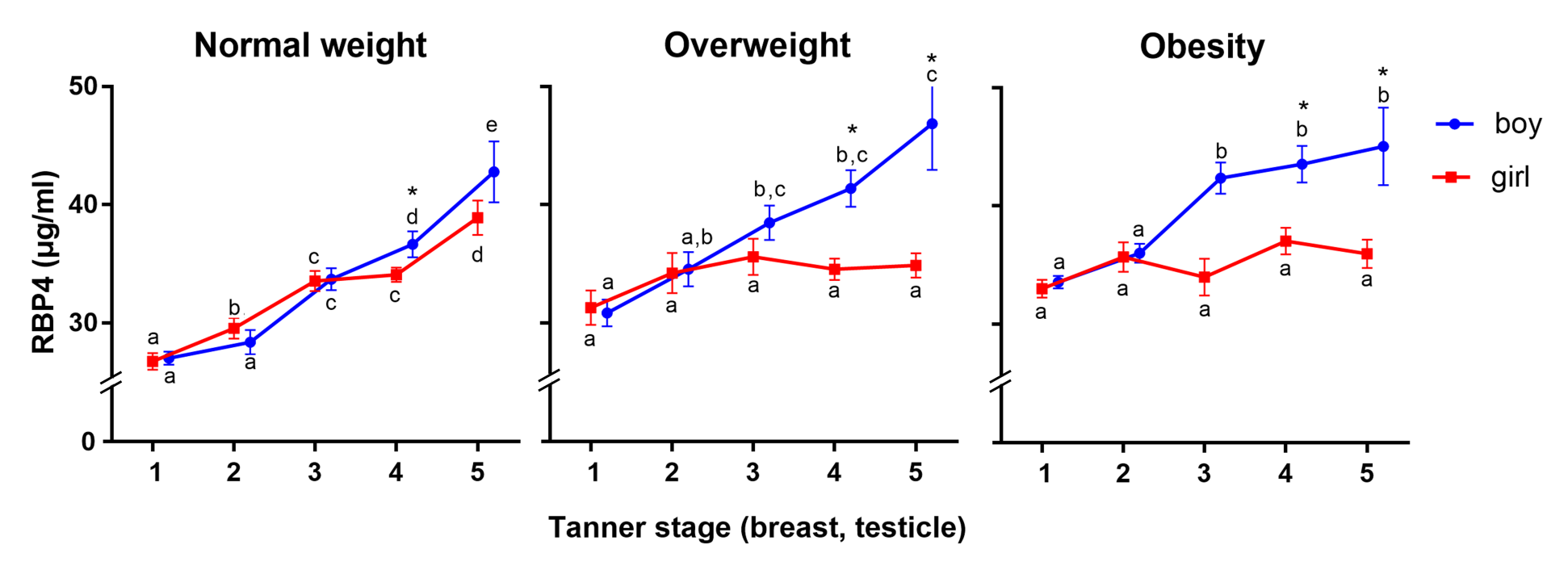
**

Fig. S2 Gender- and puberty- differences in serum RBP4 levels. Data are displayed as mean ± SE. The difference between boys and girls in the same tanner group was evaluated by independent-sample T tests with * indicating *P* < 0.05. The difference between diverse tanner groups of the same gender was evaluated by ANOVA. Statistically significant differences (*P* < 0.05) in RBP4 levels between tanner groups are indicated by different letters, whereas differences between RBP4 levels in groups of the same gender with the same letter are not statistically significant.

| Table S1. Baseline and follow-up characteristics of subjects in the prospective study | | |
| --- | --- | --- |
| Variables | At baseline | At follow-up |
| N | 352 | 352 |
| Age (year) | 11.9 ± 3.1 | 20.7 ± 2.8 |
| Gender (male %) | 51.1 | 50.6 |
| Puberty (stage 1/2/3/4/5 %) | 33.6/12.7/11.2/25.1/17.4 | - |
| Exercise ≥ 3 times/week (%) | 55.6 | - |
| Dietary score | 27.9 ± 4.5 | - |
| BMI (kg/m^2^) | 22.5 ± 4.8 | 26.23 ± 5.74 |
| WC (cm) | 74.1 ± 12.7 | 86.5 ± 14.7 |
| Body Fat percentage ( % ) | 25.7 ± 8.3 | 31.6 ± 10.4 |
| SBP (mm Hg) | 108 ± 13 | 115.5 ± 14.4 |
| DBP (mm Hg) | 68 ± 10 | 74.1 ± 10.4 |
| TG (mmol/L) | 1.12 ± 0.55 | 1.21 ± 0.98 |
| TC (mmol/L) | 4.16 ±0.79 | 4.56 ± 2.37 |
| HDL-C (mmol/L) | 1.40 ± 0.31 | 1.45 ± 0.34 |
| LDL- C (mmol/L) | 2.59 ± 0.73 | 2.60 ± 0.73 |
| Glucose (mmol/L) | 5.13 ± 0.52 | 4.96 ± 0.93 |
| OGTT 2h glucose (mmol/L) | - | 6.09 ± 1.99 |
| HbA1c (%) | - | 5.40 ± 0.66 |
| Insulin (mU/L) | 8.49 (5.23-13.13) | 7.25 (4.07-11.73) |
| HOMA-IR | 1.93 (1.15-2.97) | 1.56 (0.88-2.56) |
| ISI_M_ | - | 5.95 (3.82-9.19) |
| MS (%) | 15.0 | 16.1 |

Abbreviations: BMI, body mass index; WC, waist circumference; SBP, systolic blood pressure; DBP, diastolic blood pressure; TG, triglycerides; TC, total cholesterol; HDL-C, high-density lipoprotein cholesterol; LDL-C, low-density lipoprotein cholesterol; HOMA-IR, homeostatic model assessment of insulin resistance; IR, insulin resistance; MS, metabolic syndrome.

All values are reported as mean ± SD, median (interquartile range) or percentage.

Table S2. Baseline characteristics of subjects with or without loss to follow-up

| Variables | Subjects without follow-up | Subjects with follow-up | *P* |
| --- | --- | --- | --- |
| N | 3,093 | 352 |  |
| Gender (male %) | 51.0 | 51.1 | 0.955 |
| Age (year) | 11.9 ± 3.1 | 11.5 ± 3.1 | **0.023** |
| Puberty (stage 1/2/3/4/5 %) | 29.0/14.6/14.1/  23.7/18.5 | 33.6/12.7/11.2/  25.1/17.4 | 0.162 |
| Exercise ≥ 3 times/week (%) | 57.0 | 55.6 | 0.615 |
| Dietary score | 27.7 ± 4.3 | 27.9 ± 4.5 | 0.485 |
| BMI (kg/m^2^) | 21.8 ± 4.9 | 22.5 ± 4.8 | **0.012** |
| WC (cm) | 72.2 ± 13.1 | 74.1 ± 12.7 | **0.010** |
| Body fat percentage ( % ) | 24.2 ± 8.6 | 25.7 ± 8.3 | **0.003** |
| SBP (mm Hg) | 108 ± 14 | 108 ± 13 | 0.737 |
| DBP (mm Hg) | 68 ± 10 | 68 ± 10 | 0.649 |
| TG (mmol/L) | 1.02 ± 0.55 | 1.12 ± 0.55 | **0.001** |
| TC (mmol/L) | 4.08 ± 0.83 | 4.16 ±0.79 | 0.083 |
| HDL-C (mmol/L) | 1.40 ± 0.32 | 1.40 ± 0.31 | 0.971 |
| LDL- C (mmol/L) | 2.54 ± 0.76 | 2.59 ± 0.73 | 0.212 |
| Glucose (mmol/L) | 5.08 ± 0.56 | 5.13 ± 0.52 | 0.097 |
| Insulin (mU/L) * | 8.49 (5.23-13.13) | 9.61 (6.01-14.65) | **< 0.001** |
| HOMA-IR* | 1.93 (1.15-2.97) | 2.16 (1.37-3.42) | **<0.001** |
| Adiponectin (μg/L)* | 5.18 (3.85-7.37) | 5.18 (3.85-7.37) | 0.223 |
| Leptin (ng/ml)* | 6.01 (1.95-13.88) | 8.12 (2.92-17.84) | **< 0.001** |
| RBP4 (μg/ml) | 34.07 ± 11.56 | 34.42 ±10.78 | 0.448 |
| MS % | 11.8 | 15.0 | 0.078 |

Abbreviations: BMI, body mass index; WC, waist circumference; SBP, systolic blood pressure; DBP, diastolic blood pressure; TG, triglycerides; TC, total cholesterol; HDL-C, high-density lipoprotein cholesterol; LDL-C, low-density lipoprotein cholesterol; HOMA-IR, homeostatic model assessment of insulin resistance; IR, insulin resistance; MS, metabolic syndrome.

All values are reported as mean ± SD, median (interquartile range) or percentage.

*Variables were ln-transformed before analysis. *P* values were calculated from two-sample t test for continuous variables or Chi-square test was used for categorical variables.
